# Histopathological grading affects survival in patients with isocitrate dehydrogenase-wildtype gliomas

**DOI:** 10.3389/fonc.2025.1570941

**Published:** 2025-09-16

**Authors:** Ziming Hou, Dongyuan Liu, Zhe Hou, Hongbing Zhang, Hao Wang

**Affiliations:** Department of Neurosurgery, Beijing Luhe Hospital, Capital Medical University, Beijing, China

**Keywords:** histopathological grade, IDH-wildtype, survival, glioma, Chinese Glioma Genome Atlas

## Abstract

**Background:**

The World Health Organization (WHO) Classification of Tumors of the Central Nervous System (2021) defines lower-grade (WHO grade II/III) isocitrate dehydrogenase (IDH) wild-type astrocytoma as glioblastoma, IDH-wildtype, WHO grade 4. However, this definition is conditional. Notably, the traditional histopathological grade is no longer used, and the independent prognostic factor of tumor grade in IDH wild-type gliomas remains unclear. In this study, we aimed to determine if histopathological grade is an independent prognostic factor.

**Methods:**

The clinical data and pathological information of 647 patients with IDH wild-type gliomas from the Chinese Glioma Genome Atlas (CGGA) database (2006-2019) were retrospectively analyzed. All patients were stratified according to histopathological grade and its prognostic significance in IDH wild-type gliomas. Univariate and Cox’s multivariate analyses were used to determine the prognostic significance.

**Results:**

The median follow-up time was 100.4 months, and the median survival time was 20.3 months. The histopathological grade was an important independent prognostic factor in the univariate and multivariate analyses, and a higher grade was associated with poor overall survival and progression-free survival. After further stratification by the extent of resection and postoperative adjuvant treatment, the histopathological grade remained a significant prognostic factor.

**Conclusions:**

In this study, histopathological grade affected survival in IDH-wild-type gliomas. This effect appears to be independent of the extent of resection and postoperative treatment. Thus, we suggest that clinical treatment of patients with IDH wild-type gliomas should continue to consider histopathological grade along with the molecular characteristics of the tumors.

## Introduction

Gliomas are the most common primary tumors of the central nervous system, with an estimated annual incidence of 6.6 per 100,000 individuals in the USA (1). Gliomas are highly invasive, difficult to completely resect, recur easily after surgery, and are difficult to cure radically. According to the 2016 World Health Organization (WHO) classification of Central Nervous System (CNS) tumors, lower-grade gliomas are divided into isocitrate dehydrogenase (IDH)-mutant gliomas and IDH-wildtype gliomas. In 2021, the fifth edition of the WHO Classification of Tumors of the CNS updated the classification of gliomas (2). Lower-grade IDH-wildtype gliomas defined in the 2016 classification are conditionally reclassified as glioblastomas in the fifth edition, and were recommended to treat these tumors according to higher malignancy glioblastoma. This classification aims to emphasize the role of molecular pathology in CNS tumor classification. Although some molecular biomarkers (such as IDH and 1p/19q) strongly affect survival (3), there are arguments regarding histopathological grade’s independent prognostic value.

Some studies have shown that lower-grade (WHO grade II-III) IDH-wildtype astrocytomas have molecular features and clinical outcomes similar to glioblastoma (4, 5). The cIMPACT-NOW has suggested that lower grade IDH-wildtype astrocytomas carrying epidermal growth factor receptor (EGFR) amplification and accompanied whole chromosome 7 gain and whole chromosome 10 loss (+7/−10) and/or telomerase reverse transcriptase (TERT) promoter mutation should be classified as glioblastomas, because of their shorter survival time (4, 6). Therefore, the 2021 WHO classification updated the criteria for a diagnosis of Glioblastoma, IDH-wildtype, WHO grade 4. The lower grade IDH-wildtype astrocytomas that met the molecular criteria for glioblastoma were classified as glioblastomas. The histopathological grade was no longer applicable for IDH-wildtype gliomas.

However, one previous study showed that histological grade was still an important prognostic factor for IDH-wildtype gliomas and suggested to be cautious when classifying IDH-wildtype grade II astrocytomas as glioblastomas, especially those with isolated TERT promoter mutation (7). Another study agrees with this view, suggesting that strictly defining IDH-wildtype grade II astrocytomas with TERT promoter mutation in isolation seemed insufficient to hypothesize that the tumor will manifest as glioblastoma, IDH-wildtype (8).

Therefore, whether the histopathological grade is an independent prognostic factor remains unclear. To clarify this issue, we reviewed the Chinese Glioma Genome Atlas (CGGA) database (2006-2019) for clinical and survival information of patients with newly diagnosed grade II-III-IV IDH-wildtype gliomas. This study aimed to determine the prognostic value of the histopathological grade in a series of consecutive patients with IDH wild-type gliomas.

This article reviews the different histopathological levels of IDH wild-type gliomas and analyzes the prognostic characteristics of different histopathological types of IDH wild-type gliomas using clinical and follow-up data. It also clarifies whether histopathology remains an independent factor affecting prognosis and clinical features that should be considered in postoperative treatment.

## Materials and methods

### Patients

We retrospectively reviewed the medical records of 647 consecutive patients with grade II, III, and IV IDH wild-type gliomas who underwent surgical resection at a single large academic medical center between 2006 and 2019. We collected information on the clinical, radiological, histological, and molecular features of the patients from the Chinese Glioma Genome Atlas database (CGGA, http://www.cgga.org.cn) (9). This database and this study have been approved by the ethics committee of Beijing Tiantan Hospital, and informed consent was obtained from all patients.

We included patients who (1) were aged ≥18 years; (2) had undergone their first surgical resection; (3) had a histological diagnosis of IDH-wild-type gliomas (grade II, III, and IV); (4) had available information on clinical and survival; and (5) had no previous adjuvant treatment.

The extent of resection (EOR) was calculated by comparing postoperative MR images with preoperative MR images. If the tumor was enhanced on preoperative MR, gross total resection (GTR) of the tumor was defined as resection without residual enhanced tumor. If the tumor was not enhanced or was partially enhanced on preoperative MR, resection was evaluated based on the residual high-intensity lesion on T2-weighted or very low-intensity lesion on T1-weighted MR. EOR was classified as gross total resection (GTR; no imaging evidence of residual tumor) or non-gross total resection (non-GTR; residual tumor). Patients who underwent a biopsy were excluded from the study.

### Molecular analysis

We obtained information on molecular pathology from the CGGA database. Brain tissue samples obtained during surgery were tested for IDH mutations and methylguanine-DNA methyltransferase (MGMT) promoter methylation. The status of IDH mutations and MGMT promoter methylation was tested using DNA pyrosequencing, as previously reported (10, 11).

### Statistical analysis

We used the chi-square test or Fisher’s exact test to test categorical variables for differences in clinical variables between the subgroups. When our Chi-square assumptions were violated, we analyzed categorical data using Fisher’s exact test. Overall survival (OS) was calculated from the date of the first surgery until death or last follow-up. Progression-free survival (PFS) was calculated from the date of the first surgery until the date of clinical or radiological progression according to the RANO criteria. OS/PFS was computed using the Kaplan-Meier method, and between-group differences in survival were tested using the log-rank test. Additionally, multivariate survival analysis of certain variables (age at diagnosis, sex, pre-operative KPS score, EOR, histopathological grade, MGMT methylation status, and treatment after surgery) were performed using the Cox proportional hazards model, and the proportional hazards assumption was tested. Statistical significance was set at p < 0.05. significant. All analyses were conducted using R statistical software R, version 3.5.1) and GraphPad Prism 8.3.

## Results

### Patients’ characteristics

**Figure 1:**
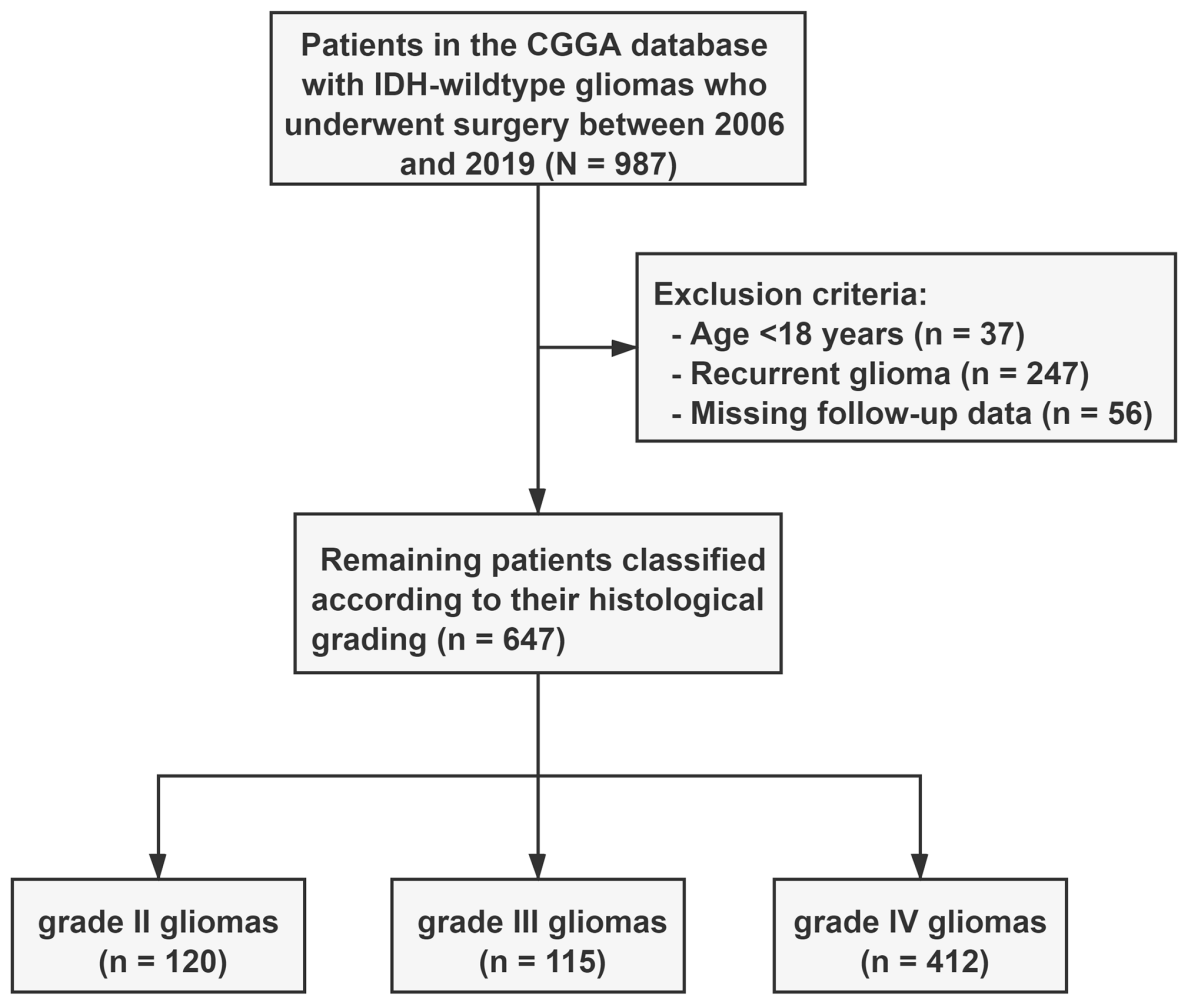
Flow chart of the 647 included patients. Patients with IDH-wildtype gliomas were classified into three subgroups based on histopathological grading. WHO, World Health Organization; CGGA, Chinese Glioma Genome Atlas.

We identified 987 patients with IDH-wildtype gliomas who underwent surgical resection from the CGGA database. Of these, 340 were excluded from the study cohort because they did not meet the inclusion criteria. Specifically, 37 patients were excluded because they were under the age of 18 years, 247 because of recurrent gliomas, and 56 because of lack of follow-up information (**Figure 1**). Consequently, our analysis focused on 647 patients with IDH wild-type gliomas; their clinical characteristics are detailed in **Table 1**. Based on the histopathological grade, the distribution revealed 120 patients (18.5%) had grade II gliomas, 115 (17.8%) had grade III gliomas, and 427 (63.7%) had grade IV gliomas.

**Table 1:** Baseline patient characteristics of all patients with IDH-wildtype glioma.

Characteristics	All patients		grade II		grade III		*p-*value*	grade IV		*p*-value**
	N	%	N	%	N	%		N	%	
Patients (n)	647	100	120	18.5	115	17.8		412	63.7	
Sex							0.230			0.782
Male	390	60.3	76	63.3	64	55.7		250	60.7	
Female	257	39.7	44	36.7	51	44.3		162	39.3	
Age, years							0.035			<0.0001
Median (IQR)	50	(40–58)	42	(32–49)	44	(35–55)		53	(45–60)	
18–50 year	343	53.0	95	79.2	77	67.0		171	41.5	
year	304	47.0	25	20.8	38	33.0		241	58.5	
Presenting symptom							<0.0001			<0.0001
Epilepsy	186	28.7	74	61.7	44	38.3		68	16.5	
Incidental	23	3.6	11	9.2	5	4.3		7	1.7	
Headache	377	58.3	39	32.5	70	60.9		268	65.0	
Miscellaneous neurologic complaints	243	37.6	23	19.2	36	31.3		184	44.7	
Preoperative KPS							0.003			<0.0001
Median (IQR)	90	(80–90)	90	(90–90)	90	(80–90)		90	(80–90)	
>80	366	56.6	92	76.7	67	58.3		207	50.2	
≤80	281	43.4	28	23.3	48	41.7		2056	49.8	
Tumor location							0.633			<0.0001
Frontal	303	46.8	74	61.7	59	51.3		170	41.3	
Temporal	305	47.1	51	42.5	58	50.4		196	47.6	
Parietal	143	22.1	16	13.3	18	15.7		109	26.5	
Insular	107	16.5	27	22.5	23	20.0		57	13.8	
Other***	289	31.4	43	29.2	46	40.0		200	48.5	
Side of lesion										
Right	303	46.8	56	46.7	54	47.0		193	46.8	
Left	305	47.1	54	45.0	55	47.0		196	47.6	
Bilateral	39	6.0	10	8.3	6	6.1		23	5.6	
EOR							0.041			0.010
GTR	240	37.1	44	36.7	28	24.3		168	40.8	
Non-GTR	407	62.9	76	63.3	87	75.7		244	59.2	
MGMT status							0.008			0.029
Methylated	247	42.4	23	26.1	45	44.5		179	45.5	
Unmethylated	335	57.6	65	73.9	56	55.5		214	54.5	
Unknown	65	/	32	/	14	/		19	/	
Treatment after surgery							<0.0001			<0.0001
Chemotherapy	36	6.0	6	5.3	6	5.5		24	6.4	
Radiotherapy	111	18.6	46	40.7	18	16.5		47	12.5	
Chemo-radiation	385	64.5	39	34.5	75	68.8		271	72.3	
Surveillance	65	10.9	22	19.5	10	9.2		33	8.8	
Unknown	50	/	7	/	6	/		35	/	

IDHmt, IDH mutant; IDHwt, IDH wild-type; IQR, interquartile range; KPS, Karnofsky Performance Score; EOR, extent of resection; GTR, gross total resection; MGMT, promoter region of the DNA repair enzyme O6-methylguanine-DNA methyltransferase.

*Comparison between grade II *vs* grade III.

** Comparison between grade II + III *vs* grade IV.

*** Basal ganglia, corpus callosum, hippocampus, thalamus, and other midline structures. P <0.05 are indicated in bold.

Of the 647 patients, 390 (60.3%) were male, and 257 (29.7%) were female. The median age at diagnosis was 50 years (interquartile range [IQR], 40–58 years). The predominant preoperative symptoms were headache (58.3%) and various neurological complaints (37.6%). The median preoperative Karnofsky Performance Status (KPS) score was 90 (IQR, 80–90). Notably, gross total resection was performed in 240 patients (37.1%), whereas 407 patients (62.9%) underwent non-gross total resection. The MGMT promoter was methylated in 247 patients (42.4%) and unmethylated in 335 patients (57.6%). Treatment after surgery modalities varied, with 36 patients (5.6%) opting for chemotherapy alone, 111 patients (17.2%) undergoing radiotherapy alone, 386 patients (59.7%) receiving chemo-radiation, and 65 patients (10.0%) adopting a ‘Wait and Scan’ approach for IDH-wildtype gliomas.

The two patient groups stratified according to histopathological grade (grades II and III) exhibited significant differences in various parameters. These included age (P = 0.035), presenting symptoms (P < 0.0001), pre-operative Karnofsky Performance Status (KPS) (P = 0.003), tumor location (P < 0.0001), extent of resection (P = 0.041), MGMT status (P = 0.008), and treatment after surgery (P < 0.0001) (**Table 1**). Furthermore, the patient groups with grade III and IV gliomas displayed statistical heterogeneity in terms of age (P < 0.0001), presenting symptoms (P < 0.0001), tumor location (P = 0.036), and extent of resection (P = 0.001) (**Supplementary Table S1**).

### Survival analysis

**Figure 2:**
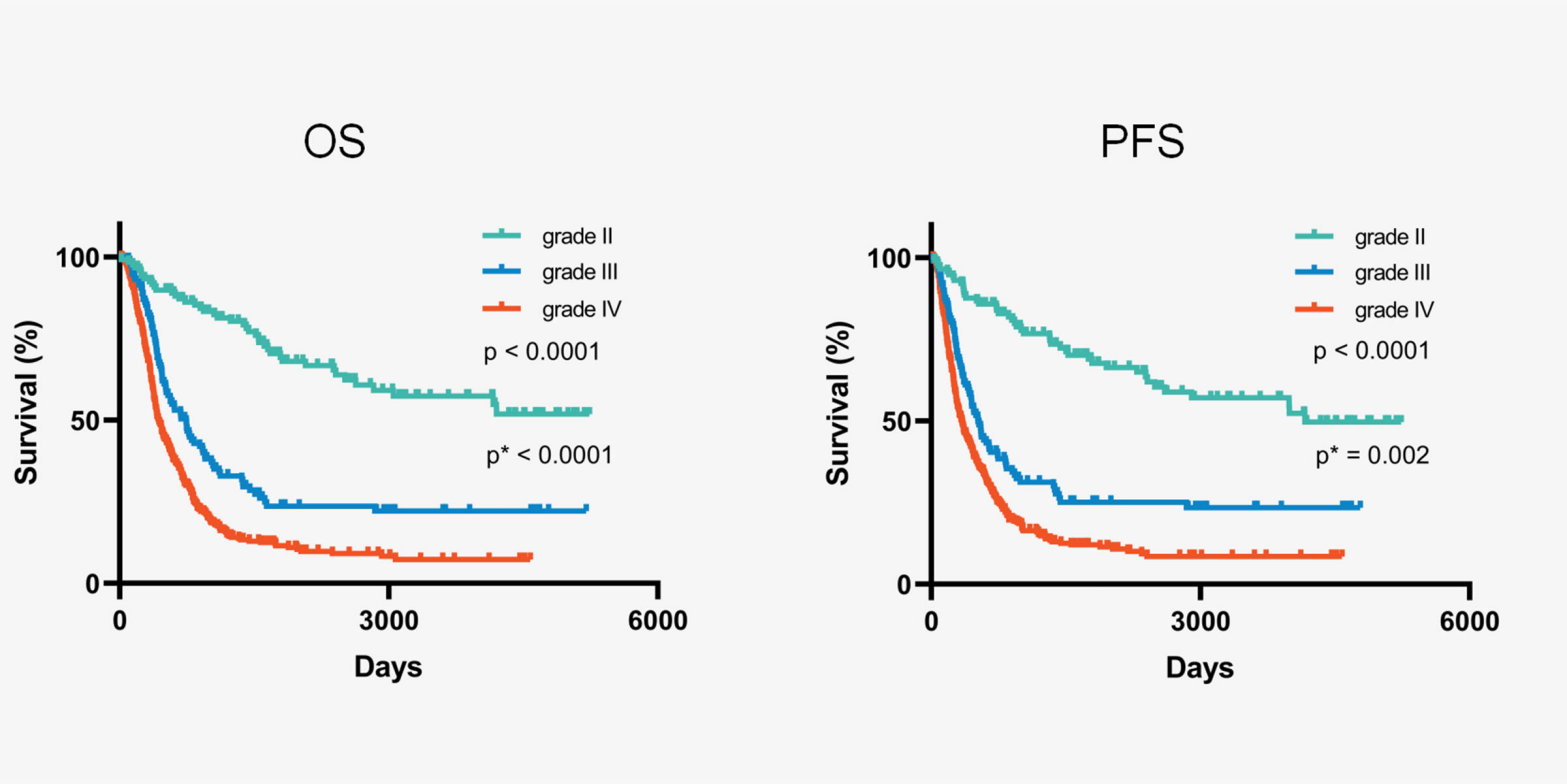
Kaplan-Meier curves of all IDH-wildtype gliomas stratified by histopathological grading. * Comparation between grade III and grade IV.

Within the cohort of 647 patients diagnosed with IDH-wildtype gliomas, the median follow-up time was 100.4 months, and the median survival was 20.3 months. In the analysis, 468 patients died, while 179 (27.7%) were either still alive or lost to follow-up. No surgery-related mortality was observed. Stratifying by histopathological grade, patients with grade IV IDH-wildtype gliomas exhibited a significantly shorter median overall survival (OS) of 14.4 months, in contrast to grade III (24.7 months) and grade II (not reached) (3-group comparison; p < 0.0001, grade II *vs*. grade III; p < 0.0001, grade III *vs*. grade IV; p < 0.0001) (**Figure 2**).

Several factors were significantly associated with OS in the univariate survival analysis of the entire cohort. These included age at diagnosis (>50 years *vs*. ≤50 years; HR, 1.914; P < 0.0001), preoperative Karnofsky Performance Status (KPS) (≤ 80 *vs*. > 80; HR, 1.564; p < 0.0001), extent of resection (non-GTR *vs*. GTR; HR, 2.267; p < 0.0001), and histopathological grade (grade III *vs*. grade II; HR, 3.268; p < 0.0001, grade IV *vs*. grade II; HR, 4.942; p < 0.0001) (refer to **Table 2**). In the subsequent multivariate analysis of the entire cohort, age at diagnosis (>50 years *vs*. ≤50 years; HR, 1.327; P = 0.007), extent of resection (non-GTR *vs*. GTR; HR, 3.295; P < 0.0001), histopathological grade (grade III *vs*. grade II; HR, 3.406; P < 0.0001, grade IV *vs*. grade II; HR, 7.964; P < 0.0001), and treatment after surgery (Chemo-radiation *vs*. other treatment; HR, 1.597; P < 0.0001) were identified as significant prognostic factors for overall survival (**Table 3**).

**Table 2:** Univariate survival analysis of all patients with IDH-wildtype glioma.

Variables	OS			PFS		
	HR	95% CI	*p*-value	HR	95% CI	*p*-value
Age, years (>50 *vs ≤*50)	1.914	1.587–2.307	<0.0001	1.813	1.495–2.200	<0.0001
Sex (male *vs* female)	1.159	0.964–1.394	0.1223	1.180	0.9751–1.429	0.0946
Pre-operation KPS (≤80 *vs >*80)	1.564	1.296–1.887	<0.0001	1.483	1.222–1.801	<0.0001
EOR (non-GTR *vs* GTR)	2.267	1.891–2.717	<0.0001	2.047	1.698–2.469	<0.0001
grade III *vs* grade II	3.268	2.276–4.693	<0.0001	3.127	2.170–4.506	<0.0001
grade IV *vs* grade II	4.942	4.022–6.074	<0.0001	4.641	3.753–5.739	<0.0001
MGMT (Unmethylated *vs* Methylated)	1.077	0.889–1.305	0.4417	1.031	0.846–1.256	0.7642
Treatment after surgery (Chemo-radiation *vs* other treatment)	1.097	0.901–1.336	0.3588	1.211	0.990–1.481	0.0679

OS, overall survival; PFS, progression free survival; HR, hazard ratio; CI, confidence interval; KPS, Karnofsky Performance Score; EOR, extent of resection; GTR, gross total resection; MGMT, promoter region of the DNA repair enzyme O6-methylguanine-DNA methyltransferase.

**Table 3:** Multivariate Cox regression of survival in all patients with IDH-wildtype glioma.

Variables	OS			PFS		
	HR	95% CI	*p*-value	HR	95% CI	*p*-value
Age, years (>50 *vs* 18–50)	1.327	1.079–1.633	0.007	1.282	1.039–1.583	0.021
EOR (non-GTR *vs* GTR)	3.295	2.627–4.132	<0.0001	2.655	2.123–3.322	<0.0001
grade III *vs* grade II	3.406	2.225–5.216	<0.0001	3.143	2.049–4.819	<0.0001
grade IV *vs* grade II	7.964	5.339–11.88	<0.0001	6.511	4.376–9.69	<0.0001
Treatment after surgery (Chemo-radiation *vs* other treatment)	1.597	1.288-1.981	<0.0001	1.477	1.181–1.849	0.001

OS, overall survival; PFS, progression free survival; HR, hazard ratio; CI, confidence interval; EOR, extent of resection; GTR, gross total resection.

Among high-grade (grade III + IV) IDH wild-type gliomas, patients with grade III gliomas exhibited longer survival than those with grade IV gliomas (P < 0.0001). Further multivariate analysis indicated that non-GTR (hazard ratio [HR], 3.082; P < 0.0001), grade IV disease (HR, 2.421; P < 0.0001), and other treatments after surgery (HR, 1.688; P < 0.0001) were associated with poor prognosis (**Supplementary Table S2**).

**Figure 3:**
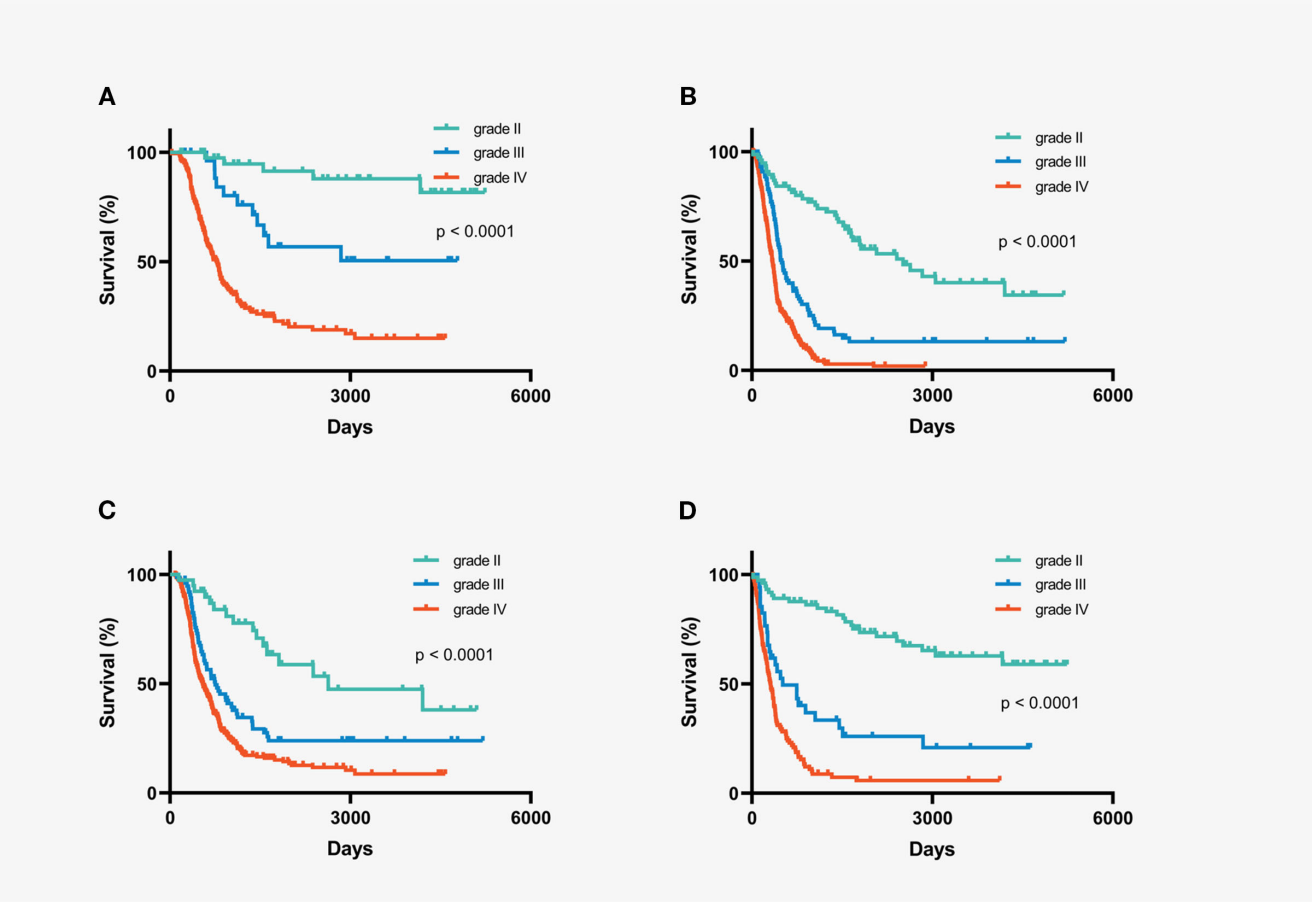
Kaplan-Meier overall survival curves of subgroups stratified by histopathological grading. **(A)** IDH-wildtype gliomas with gross total resection **(B)** IDH-wildtype gliomas with non-gross total resection **(C)** IDH-wildtype gliomas with chemo-radiation therapy after surgery **(D)** IDH-wildtype gliomas with chemotherapy or radiation or follow-up after surgery.

Extent of resection (EOR) and postoperative treatment have emerged as independent prognostic factors for IDH-wild-type gliomas. Consequently, understanding variability in treatment and its prognostic impact is crucial. To minimize the influence of EOR and postoperative treatment on survival, we analyzed the role of histopathological grade in IDH wild-type gliomas that underwent either gross total resection (GTR) or chemoradiotherapy after surgery. Notably, patients with grade II gliomas, both in the GTR and chemo-radiation subgroups, demonstrated longer survival than those with higher-grade gliomas (**Figure 3**). In multivariate analysis of the subgroups of patients who underwent GTR and postoperative chemoradiotherapy, histopathological grade emerged as an important independent prognostic factor, with low-grade gliomas associated with longer survival (**Supplementary Tables S3** and **S5**).

## Discussion

Diffuse gliomas are the most common and invasive primary brain tumors in adults. Molecular markers are significant prognostic factors that play important roles in the diagnosis and treatment of gliomas. IDH mutations and 1p/19q co-deletion are associated with longer survival, and IDH wild-type gliomas often have a poor prognosis (12, 13). In 2021, the WHO reclassified gliomas by integrating molecular and histologic characteristics (2). This classification further emphasizes the role of molecular pathology in glioma grading, and molecular markers overrule histopathological diagnosis.

The reclassification divided adult gliomas into astrocytoma, IDH mutant; oligodendroglioma, IDH mutant, and 1p/19q codeletion; and glioblastoma, IDH-wildtype. Intriguingly, grade II and III IDH-wild-type astrocytomas were conditionally classified as glioblastoma, IDH-wild-type, and WHO grade IV, rendering histopathological grading obsolete for IDH-wild-type gliomas.

However, several previous studies have revealed that the histopathological grade is an independent prognostic factor in IDH-wildtype gliomas (12, 14–16). A previous study of 558 cases of lower-grade gliomas (WHO grade II-III) revealed that the histological grade had important prognostic significance in IDH-wildtype gliomas (17). The OS of grade II IDH-wildtype gliomas (median survival time of 4.82 years) was significantly longer than that of grade III IDH-wildtype gliomas (median survival time 1.97 years). Another study also pointed out that the histopathological grade impacted the survival of IDH-wild-type gliomas (15). Univariate and multivariate survival analyses showed that high-grade gliomas were significantly associated with poorer prognosis.

In our study, we found that histopathological grade was still an important independent prognostic factor in IDH wild-type gliomas and had an important prognostic value. Moreover, there were significant differences in the clinical features among the different histological grades of IDH-wild-type gliomas. Patients with grade IV IDH wild-type gliomas were older than those with grade II and III gliomas. Preoperative KPS of patients with grade II gliomas was better than that of patients with grades III and IV gliomas. The presenting symptoms of patients with grade II gliomas are more often epilepsy, while those of patients with grade III and IV gliomas are more often headache, and the tumor-occupying effect is more obvious. Grade IV gliomas are more likely to involve deep brain structures. Our conclusions are supported by the results of recent studies.

Berzero et al. explored the prognostic significance of histopathological grading in lower-grade IDH-wildtype gliomas (7). He collected 47 astrocytoma IDH-wildtype grade II and 255 astrocytoma IDH-wildtype grade III; the median OS of grade II astrocytoma was more longer compared with that of grade III astrocytoma (59 months *vs* 19 months, P<0.0001). He then strictly screened 29 grade II astrocytomas and 166 grade III astrocytomas, meeting the definition of molecular glioblastoma according to the cIMPACT-NOW update 3. The authors found that the median OS of IDH-wild-type grade II gliomas was longer than that of IDH-wild-type grade III gliomas (42 *vs*. 17 months, P < 0.0001). In addition, IDH-wildtype grade II gliomas have a lower molecular variations burden compared with IDH-wildtype grade III gliomas. This study revealed the importance of histological grading for the prognostic stratification of IDH-wild-type gliomas.

Giannini also believed that IDH wild-type grade II glioma combined with a TERT mutation in isolation was not suitable to be defined as glioblastoma, IDH wild-type (8). Because the survival time of these patients is significantly longer, and the histopathological grade remains a useful prognostic stratification of IDH wild-type glioma, which should be carefully considered in future diagnosis, treatment, and clinical trials.

However, Taboulet et al. (18) found that histopathological grade was not a prognostic factor for high-grade (grade III and grade IV) IDH-wild-type gliomas in univariate and multivariate analyses. There was no difference in survival between patients with grade III and IV gliomas. Interestingly, our results contradict these findings. We found that grade IV gliomas were associated with worse survival, and histopathological grade was an important prognostic factor for high-grade IDH wild-type gliomas. Another study also showed that low-grade IDH-wild-type gliomas had longer survival than IDH-wild-type glioblastomas (13).

Most studies have shown that total surgical resection and postoperative chemoradiation can prolong the survival time of IDH-wildtype gliomas and are independent prognostic factors for IDH-wildtype gliomas (19, 20). Our findings also support the widely accepted notion that total surgical resection and postoperative chemoradiation independently prolong the survival of IDH-wildtype gliomas. Importantly, even when considering treatment variations, histological grade remained a robust independent prognostic factor across subgroups. Higher grades were consistently correlated with shorter survival times.

Despite the strengths of our study, such as its substantial sample size, its retrospective nature introduces a potential bias. The lack of molecular marker information for distinguishing lower-grade IDH-wild-type astrocytomas meeting the definition of molecular glioblastoma and potential biases in stratifying IDH-wild-type low-grade gliomas based solely on histopathology are acknowledged as limitations. Future research incorporating comprehensive molecular analyses and prospective designs may further refine our understanding of the prognostic landscape in IDH-wildtype gliomas.

## Conclusions

In our study, histopathological grade was still an independent prognostic factor affecting survival in IDH-wild-type gliomas. Notably, this prognostic influence remains distinct and is seemingly unaffected by the extent of surgical resection and postoperative treatment. Consequently, our findings underscore the continued importance of considering histopathological grade in both diagnosis and treatment planning for IDH wildtype gliomas, contributing to the enhanced clinical and therapeutic management of patients. This emphasis on histopathological grade is particularly relevant for guiding the design and implementation of future clinical trials, in which a comprehensive understanding of its prognostic significance is essential for advancing research and improving patient outcomes.

## Data Availability

The raw data supporting the conclusions of this article will be made available by the authors, without undue reservation.

## References

[1] Ostrom QT Gittleman H Xu J Kromer C Wolinsky Y Kruchko C. CBTRUS statistical report: primary brain and other central nervous system tumors diagnosed in the United States in 2009-2013. Neuro Oncol. (2016) 18:v1–v75. doi: 10.1093/neuonc/now207, PMID: 28475809 PMC8483569

[2] Louis DN Perry A Wesseling P Brat DJ Cree IA Figarella-Branger D. The 2021 WHO classification of tumors of the central nervous system: a summary. Neuro-Oncology. (2021) 23:1231–51. doi: 10.1093/neuonc/noab106, PMID: 34185076 PMC8328013

[3] Louis DN Perry A Reifenberger G von Deimling A Figarella-Branger D Cavenee WK. The 2016 world health organization classification of tumors of the central nervous system: a summary. Acta Neuropathol. (2016) 131:803–20. doi: 10.1007/s00401-016-1545-1, PMID: 27157931

[4] Brat DJ Aldape K Colman H Holland EC Louis DN Jenkins RB. cIMPACT-NOW update 3: recommended diagnostic criteria for “Diffuse astrocytic glioma, IDH-wildtype, with molecular features of glioblastoma, WHO grade IV. Acta Neuropathol. (2018) 136:805–10. doi: 10.1007/s00401-018-1913-0, PMID: 30259105 PMC6204285

[5] Tesileanu CMS Dirven L Wijnenga MMJ Koekkoek JAF Vincent AJPE Dubbink HJ. Survival of diffuse astrocytic glioma, IDH1/2 wildtype, with molecular features of glioblastoma, WHO grade IV: a confirmation of the cIMPACT-NOW criteria. Neuro-oncology. (2020) 22:515–23. doi: 10.1093/neuonc/noz200, PMID: 31637414 PMC7158657

[6] Louis DN Wesseling P Aldape K Brat DJ Capper D Cree IA. cIMPACT-NOW update 6: new entity and diagnostic principle recommendations of the cIMPACT-Utrecht meeting on future CNS tumor classification and grading. Brain Pathol. (2020) 30:844–56. doi: 10.1111/bpa.12832, PMID: 32307792 PMC8018152

[7] Berzero G Di Stefano AL Ronchi S Bielle F Villa C Guillerm E. IDH-wildtype lower grade diffuse gliomas: the importance of histological grade and molecular assessment for prognostic stratification. Neuro-Oncology. (2020) 23:955–66. doi: 10.1093/neuonc/noaa258, PMID: 33173941 PMC8168809

[8] Giannini C Giangaspero F. TERT promoter mutation: is it enough to call a WHO grade II astrocytoma IDH wild-type glioblastoma? Neuro Oncol. (2021) 23:865–6. doi: 10.1093/neuonc/noab052, PMID: 33660766 PMC8168806

[9] Zhao Z Zhang K-N Wang Q Li G Zeng F Zhang Y. Chinese glioma genome atlas (CGGA): A comprehensive resource with functional genomic data from chinese glioma patients. Genom Proteomics Bioinf. (2021) 19:1–12. doi: 10.1016/j.gpb.2020.10.005, PMID: 33662628 PMC8498921

[10] Wei Y Wei Z Gan Y Zhaoshi B Yongzhi W Yanwei L. Correlation of IDH1 mutation with clinicopathologic factors and prognosis in primary glioblastoma: a report of 118 patients from China. PloS One. (2012) 7:e30339. doi: 10.1371/journal.pone.0030339, PMID: 22291938 PMC3264567

[11] Hu H Wang Z Liu Y Zhang C Li M Zhang W. Genome-wide transcriptional analyses of Chinese patients reveal cell migration is attenuated in IDH1-mutant glioblastomas. Cancer Lett. (2015) 357:566–74. doi: 10.1016/j.canlet.2014.12.018, PMID: 25511738

[12] Wiestler B Capper D Sill M Jones DTW Hovestadt V Sturm D. Integrated DNA methylation and copy-number profiling identify three clinically and biologically relevant groups of anaplastic glioma. Acta Neuropathol. (2014) 128:561–71. doi: 10.1007/s00401-014-1315-x, PMID: 25008768

[13] Cancer Genome Atlas Research N Brat DJ Verhaak RG Aldape KD Yung WK Salama SR. Comprehensive, integrative genomic analysis of diffuse lower-grade gliomas. N Engl J Med. (2015) 372:2481–98. doi: 10.1056/NEJMoa1402121, PMID: 26061751 PMC4530011

[14] Suzuki H Aoki K Chiba K Sato Y Shiozawa Y Shiraishi Y. Mutational landscape and clonal architecture in grade II and III gliomas. Nat Genet. (2015) 47:458–68. doi: 10.1038/ng.3273, PMID: 25848751

[15] Pekmezci M Rice T Molinaro AM Walsh KM Decker PA Hansen H. Adult infiltrating gliomas with WHO 2016 integrated diagnosis: additional prognostic roles of ATRX and TERT. Acta Neuropathol. (2017) 133:1001–16. doi: 10.1007/s00401-017-1690-1, PMID: 28255664 PMC5432658

[16] Aibaidula A Chan AK-Y Shi Z Li Y Zhang R Yang R. Adult IDH wild-type lower-grade gliomas should be further stratified. Neuro-oncology. (2017) 19:1327–37. doi: 10.1093/neuonc/nox078, PMID: 28575485 PMC5596181

[17] Olar A Wani KM Alfaro-Munoz KD Heathcock LE van Thuijl HF Gilbert MR. IDH mutation status and role of WHO grade and mitotic index in overall survival in grade II-III diffuse gliomas. Acta Neuropathol. (2015) 129:585–96. doi: 10.1007/s00401-015-1398-z, PMID: 25701198 PMC4369189

[18] Tabouret E Nguyen AT Dehais C Carpentier C Ducray F Idbaih A. Prognostic impact of the 2016 WHO classification of diffuse gliomas in the French POLA cohort. Acta Neuropathol. (2016) 132:625–34. doi: 10.1007/s00401-016-1611-8, PMID: 27573687

[19] Gessler F Bernstock JD Braczynski A Lescher S Baumgarten P Harter PN. Surgery for glioblastoma in light of molecular markers: impact of resection and MGMT promoter methylation in newly diagnosed IDH-1 wild-type glioblastomas. Neurosurgery. (2019) 84:190–7. doi: 10.1093/neuros/nyy049, PMID: 29617848 PMC6500906

[20] Delev D Heiland DH Franco P Reinacher P Mader I Staszewski O. Surgical management of lower-grade glioma in the spotlight of the 2016 WHO classification system. J Neurooncol. (2019) 141:223–33. doi: 10.1007/s11060-018-03030-w, PMID: 30467813

