# Supplementary material for: Histopathological grading affects survival in patients with isocitrate dehydrogenase-wildtype gliomas
Source: Front Oncol. 2025 Sep 16;15:1570941. doi: 10.3389/fonc.2025.1570941 (PMC12479321; doi:10.3389/fonc.2025.1570941)
Supplement: Supplementary file 1 [file Table1.doc]

**Supplementary Materials**

Table S1. Baseline patient characteristics of all patients with grade III+IV IDH-wildtype glioma

| **Characteristics** |  | **grade III** | |  | **grade IV** | | ***p*-value** |
| --- | --- | --- | --- | --- | --- | --- | --- |
|  |  | ***N*** | **%** |  | ***N*** | **%** |  |
| **Patients (n)** |  | 115 | 17.8 |  | 412 | 63.7 |  |
| **Sex** |  |  |  |  |  |  | 0.331 |
| Male |  | 64 | 55.7 |  | 250 | 60.7 |  |
| Female |  | 51 | 44.3 |  | 162 | 39.3 |  |
| **Age, years** |  |  |  |  |  |  | **<0.0001** |
| Median (IQR) |  | 44 | (35–55) |  | 53 | (45–60) |  |
| 18–50 year |  | 77 | 67.0 |  | 171 | 41.5 |  |
| >50 year |  | 38 | 33.0 |  | 241 | 58.5 |  |
| **Presenting symptom** |  |  |  |  |  |  | **<0.0001** |
| Epilepsy |  | 44 | 38.3 |  | 68 | 16.5 |  |
| Incidental |  | 5 | 4.3 |  | 7 | 1.7 |  |
| Headache |  | 70 | 60.9 |  | 268 | 65.0 |  |
| Miscellaneous  neurologic complaints |  | 36 | 31.3 |  | 184 | 44.7 |  |
| **Preoperative KPS** |  |  |  |  |  |  | 0.128 |
| Median (IQR) |  | 90 | (80–90) |  | 90 | (80–90) |  |
| >80 |  | 67 | 58.3 |  | 207 | 50.2 |  |
| ≤80 |  | 48 | 41.7 |  | 205 | 49.8 |  |
| **Tumor location** |  |  |  |  |  |  | **0.036** |
| Frontal |  | 59 | 51.3 |  | 170 | 41.3 |  |
| Temporal |  | 58 | 50.4 |  | 196 | 47.6 |  |
| Parietal |  | 18 | 15.7 |  | 109 | 26.5 |  |
| Insular |  | 23 | 20.0 |  | 57 | 13.8 |  |
| Other*** |  | 46 | 40.0 |  | 200 | 48.5 |  |
| **Side of lesion** |  |  |  |  |  |  |  |
| Right |  | 54 | 47.0 |  | 193 | 46.8 |  |
| Left |  | 55 | 47.0 |  | 196 | 47.6 |  |
| Bilateral |  | 6 | 6.1 |  | 23 | 5.6 |  |
| **EOR** |  |  |  |  |  |  | **0.001** |
| GTR |  | 28 | 24.3 |  | 168 | 40.8 |  |
| Non-GTR |  | 87 | 75.7 |  | 244 | 59.2 |  |
| **MGMT status** |  |  |  |  |  |  | 0.858 |
| Methylated |  | 45 | 44.5 |  | 179 | 45.5 |  |
| Unmethylated |  | 56 | 55.5 |  | 214 | 54.5 |  |
| Unknown |  | 14 | / |  | 19 | / |  |
| **Treatment after surgery** |  |  |  |  |  |  | 0.739 |
| Chemotherapy |  | 6 | 5.5 |  | 24 | 6.4 |  |
| Radiotherapy |  | 18 | 16.5 |  | 47 | 12.5 |  |
| Chemo-radiation |  | 75 | 68.8 |  | 271 | 72.3 |  |
| Surveillance |  | 10 | 9.2 |  | 33 | 8.8 |  |
| Unknown |  | 6 | / |  | 35 | / |  |

Table S2. Multivariate Cox regression of survival in all patients with grade III+IV IDH-wildtype glioma

| **Variables** | **OS** | | |  | **PFS** | | |
| --- | --- | --- | --- | --- | --- | --- | --- |
|  | **HR** | **95% CI** | ***p*-value** |  | **HR** | **95% CI** | ***p*-value** |
| **EOR (non-GTR vs GTR)** | 3.082 | 2.444–3.886 | **<0.0001** |  | 2.486 | 1.971-3.135 | **<0.0001** |
| **grade IV vs grade III** | 2.421 | 1.842–3.182 | **<0.0001** |  | 2.117 | 1.608-2.789 | **<0.0001** |
| **Treatment after surgery**  **(Chemo-radiation vs other treatment)** | 1.688 | 1.351–2.110 | **<0.0001** |  | 1.607 | 1.270-2.034 | **<0.0001** |
| **MGMT status (unmethylation vs methylation)** |  |  |  |  | 1.246 | 1.005-1.545 | **0.045** |

Table S3. Multivariate Cox regression of survival in patients with Chemo-radiation treatment after surgery

| **Variables** | **OS** | | |  | **PFS** | | |
| --- | --- | --- | --- | --- | --- | --- | --- |
|  | **HR** | **95% CI** | ***p*-value** |  | **HR** | **95% CI** | ***p*-value** |
| **Age, years (>50 vs 18–50)** | 1.313 | 1.022–1.686 | **0.033** |  | – | – | – |
| **Sex,** | 0.772 | 0.599–0.994 | **0.045** |  | – | – | – |
| **grade III vs grade II** | 2.709 | 1.462–5.017 | **0.002** |  | 2.864 | 1.548–5.297 | **0.001** |
| **grade IV vs grade II** | 5.558 | 3.102–9.958 | **<0.0001** |  | 5.171 | 2.903–9.214 | **<0.0001** |
| **EOR (non-GTR vs GTR)** | 2.75 | 2.106–3.592 | **<0.0001** |  | 2.22 | 1.707–2.887 | **<0.0001** |

Table S4. Multivariate Cox regression of survival in patients with other treatment after surgery

| **Variables** | **OS** | | |  | **PFS** | | |
| --- | --- | --- | --- | --- | --- | --- | --- |
|  | **HR** | **95% CI** | ***p*-value** |  | **HR** | **95% CI** | ***p*-value** |
| **grade III vs grade II** | 3.525 | 1.893–6.565 | **<0.0001** |  | 2.76 | 1.453–5.244 | **0.002** |
| **grade IV vs grade II** | 10.205 | 5.836–17.844 | **<0.0001** |  | 8.316 | 4.775–14.483 | **<0.0001** |
| **EOR (non-GTR vs GTR)** | 4.526 | 2.861–7.158 | **<0.0001** |  | 3.66 | 2.297–5.832 | **<0.0001** |

Table S5. Multivariate Cox regression of survival in patients with gross total resection

| **Variables** | **OS** | | |  | **PFS** | | |
| --- | --- | --- | --- | --- | --- | --- | --- |
|  | **HR** | **95% CI** | ***p*-value** |  | **HR** | **95% CI** | ***p*-value** |
| **grade III vs grade II** | 3.228 | 1.01–10.318 | **0.048** |  | 2.339 | 0.85–6.44 | 0.1 |
| **grade IV vs grade II** | 9.609 | 3.512–26.289 | **<0.0001** |  | 7.15 | 3.124–16.364 | **<0.0001** |
| **Pre-operation KPS (≤80 vs >80)** | 1.48 | 1.036–2.114 | **0.031** |  | – | – | – |

Table S6. Multivariate Cox regression of survival in patients with non-gross total resection

| **Variables** | **OS** | | |  | **PFS** | | |
| --- | --- | --- | --- | --- | --- | --- | --- |
|  | **HR** | **95% CI** | ***p*-value** |  | **HR** | **95% CI** | ***p*-value** |
| **Age, years (>50 vs 18–50)** | 1.348 | 1.048–1.734 | **0.02** |  | 1.318 | 1.017–1.708 | **0.037** |
| **grade III vs grade II** | 3.462 | 2.18–5.499 | **<0.0001** |  | 3.382 | 2.106–5.432 | **<0.0001** |
| **grade IV vs grade II** | 7.372 | 4.702–11.557 | **<0.0001** |  | 6.194 | 3.93–9.761 | **<0.0001** |
| **Treatment after surgery**  **(Chemo-radiation vs other treatment)** | 1.824 | 1.42–2.342 | **<0.0001** |  | 1.693 | 1.304–2.199 | **<0.0001** |


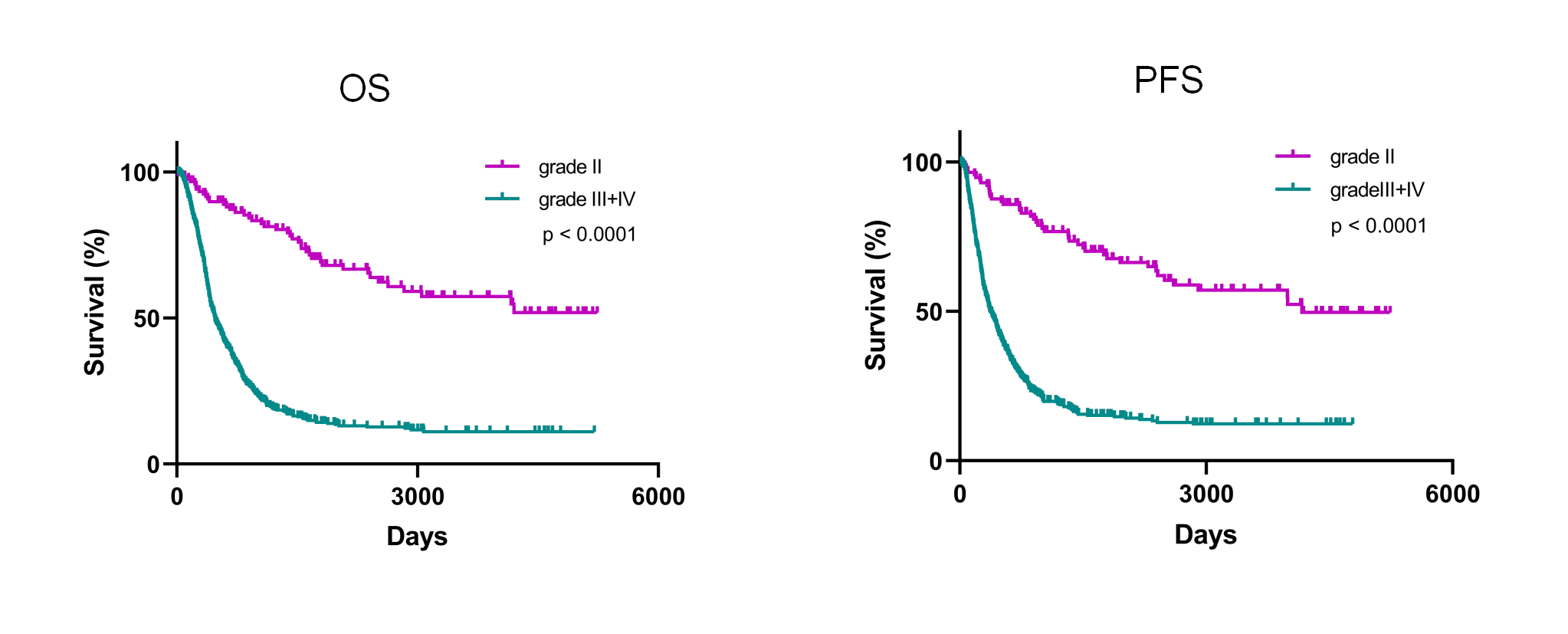


**Fig. S1** Kaplan-Meier curves of all IDH-wildtype gliomas stratified by histopathological grade.
